# Prevalence, Associated Risk Factors of Haemonchosis and Burden of Strongyle Nematode of Small Ruminants in Bishoftu, Oromia, Central Ethiopia

**DOI:** 10.1155/japr/4417948

**Published:** 2025-10-10

**Authors:** Tigist Shittu Demessie, Dereje Regassa Nigussie, Tolcha Mitiku Biru, Yihenew Getahun Ambaw

**Affiliations:** College of Veterinary Medicine, Haramaya University, Dire Dawa, Ethiopia

**Keywords:** Bishoftu, coproculture, *Haemonchus*, prevalence, sheep and goat

## Abstract

*Haemonchus contortus* is a significant blood-sucking parasitic disease. It causes economic loss through animal death and decreased production. A cross-sectional study was conducted in Bishoftu town, Oromia, Central Ethiopia, from November 2023 to May 2024 to estimate the prevalence of *H. contortus* infection and associated risk factors in shoats. A total of 384 fecal samples (262 sheep and 122 goats) were collected, with the overall prevalence of 66.9% but higher in sheep (73.3%) than goats (53.3%), females (58.6%) than males (78.5%), and in young animals. Management, age, sex, and small ruminant species were statistically significant risk factors for the prevalence of *H. contortus* in small ruminants. The mean fecal worm egg count for all small ruminants was 800 EPG. In *Haemonchus*-infected, the level of parasite burden in shoats was 58.8% (severe), 12.1% (moderate), and 29.2% (light). Small ruminant species, sex, age group, body condition, and management had statistically significant associations with the levels of strongyle egg count. *H. contortus* in shoats is a prevalent disease in Bishoftu. Therefore, it is important to implement targeted and seasonally based deworming strategies using effective anthelmintics, particularly before periods of high parasite transmission. This approach should be based on local epidemiological data to reduce parasite resistance and emphasize the susceptibility of young and female sheep and goats.

## 1. Introduction

Raising animals is a major part of small-scale Ethiopian farmers' ability to support themselves [1], with small ruminants (i.e., sheep and goats) being the most valuable farm animals. The country has around 42.9 and 52.5 million head of sheep and goats, respectively [2]. In developing countries, such as Ethiopia, sheep and goats are vital assets playing crucial roles in household economies and food security. They provide a variety of benefits for farmers, both practical and cultural. These benefits, like income from selling meat, milk, skins, and manure, can be direct and easy to quantify [3].

In the highlands of Ethiopia, where farming combines crops and animals, 11%–60% of households have small ruminants. This number is even higher, from 41% to 95%, in the lowland areas where herding is the main way of life [4]. The main reason to raise sheep and goats is that they are relatively cheap to buy and reproduce quickly and can have multiple offspring each year. This means that farmers see a return on their investment faster than they would with cattle [5]. They contribute significantly to Ethiopia's economy, making up a quarter of the value of all meat produced and bringing in foreign currency through export [6].

However, the contribution from this livestock resource is less than its potential due to several factors. From these, gastrointestinal (GI) nematode parasites, commonly known as roundworms, are a major parasitic threat in most small ruminant production systems due to the suitable geographic and climatic conditions of the country impacting productivity, growth, and survival in small ruminant production in Ethiopia [7; 8; 9].

Out of all the GI nematodes that infect sheep and goats, *Haemonchus contortus* is considered the most difficult to manage. It is common, highly pathogenic, and can quickly reproduce. In addition to this, *H. contortus* is capable of developing resistance to the drug farmers typically use for control and has ways to survive harsh conditions. These factors make it the most important parasite for sheep and goat farmers compared to other roundworms [7].

Sheep and goats infected with haemonchosis had pale mucus membranes due to the worms sucking their blood. These parasites are relentless blood feeders, taking about 0.05 mL of blood each day from an infected animal. This blood loss can lead to high death rates and hinder weight gain in affected herds [8]. In addition to haemonchosis (caused by *H. contortus*), other strongyle infections in sheep and goats are significant contributors to economic losses through reduced productivity and increased mortality. These losses are often substantial, particularly in resource-poor regions where parasitic diseases are a major constraint on small ruminant production addition to haemonchosis caused by *H. contortus* and effects from other strongyle infections in sheep and goats are significant contributors to economic losses through reduced productivity and increased mortality. These losses are often substantial, particularly in resource-poor regions where parasitic diseases are a major constraint on small ruminant production [9].

While some research has been done on haemonchosis in Bishoftu, Ethiopia [7; 13], there is a lack of recent specific data in this area on how common the infection is in sheep and goats. Understanding the current prevalence of *H. contortus* and other strongyle nematodes in the study area is crucial for developing effective prevention and control strategies. This knowledge enables the implementation of targeted interventions to mitigate impacts and enhance the health and productivity of small ruminants.

The main objectives of this study were to estimate the coproscopic prevalence of *H. contortus* of small ruminants in Bishoftu, to estimate the burden of other strongyle nematodes, and to assess associated risk factors that might contribute to infection.

## 2. Material and Methods

### 2.1. Description of Study Area

**Figure 1:**
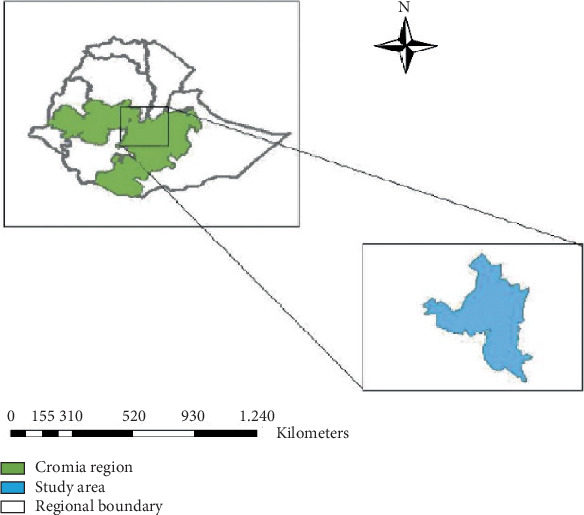
Map of Bishoftu town, East Shewa Zone, Ethiopia*. Source:* [11].

The study was conducted at Bishoftu town, Oromia, Ethiopia (8° 44⁣′ 59.99⁣^″^ N and 38° 58⁣′ 59.99⁣^″^ E), which is located in the East Shewa Zone of Oromia Regional State in Central Ethiopia, at 45 km from Addis Ababa, an altitude of 1920 m above sea level, and with average annual rainfall of 776 mm [10]. The sampled animals were from three subcities: Chalalka, Dukem, and Dhibayu (Figure 1).

### 2.2. Study Population

All small ruminants reared in both small-scale and commercial farms in Bishoftu were considered the target population, whereas small ruminants found in Dhibayu, Chalalka, and Dukem subcities were the study population.

The study focused on local sheep and goat breeds raised by smallholder farmers and commercial farms in the study area. Some smallholder households keep their animals together on communal grazing pastures during the day and separate them at night in individual enclosures with additional feed. Other smallholder farmers' animals roamed freely on communal land year-round, with no additional feeding. In contrast, commercial farms raise animals intensively for market purposes.

### 2.3. Study Design

A cross-sectional study was conducted between November 2023 and April 2024 to estimate the prevalence of *H. contortus* and burden of strongyle infection in small ruminants at a single point in time. This design is particularly useful for identifying associations between infection status and potential risk factors.

### 2.4. Sampling Method and Sample Size Determination

First, a proportionate probability sampling technique was employed for the three subcities of Bishoftu City. Locations within the subcities were purposefully chosen based on the number of animals available there. Then, a simple random sampling or lottery method was used to collect fecal samples from individual sheep and goats within smallholder households and commercial farms. The minimum number of individual sheep and goats preselected for fecal samples from smallholder households and commercial farms was taken as proportional to the total number of animals they owned.

The sample size was determined using Thrusfield's formula [12]:

 $$\begin{array}{c} \frac{N=1.96^{2}\times \text{Pexp}\left(1-\text{Pexp}\right)}{d^{2}}, \end{array}$$

where *N* is the sample size, *P* is the expected prevalence (assumed to be 50% in this case), and *d* is the desired absolute precision (at 5%).

Since there was no any prior coproscopic evidence of the prevalence of strongyle infection in the area, the assumed prevalence was set at 50% with a confidence level of 95% and a margin of error of 5%. Under these assumptions, the total sample size was 384 animals.

#### 2.4.1. Inclusion and Exclusion Criteria

The criteria for inclusion encompassed all small ruminants located in Bishoftu town throughout the study period, specifically those older than 1 month. The exclusion criteria were small ruminant owners unwilling to have samples taken from their animals.

#### 2.4.2. Study Variables

The dependent variables were the prevalence of *H. contortus* and other strongyle parasites in feces (eggs per gram [EPG]). The independent variables were species, sex, age, body condition, flock size, type of flock, management practices, sources of water, and history of deworming.

#### 2.4.3. Operational Definition

Flock sizes were categorized as small (1–5), medium (5–10), semilarge (11–20), and large (> 20) based on the number of sheep and goats per flock [13].

This study followed the age group classification system used by [14]. Animals less than 1 year old were categorized as young, and those greater than 1 year old were considered adults. To estimate the age of each animal, a method based on tooth eruption was described by [15].

Additionally, the body condition of the animals was assessed using a scoring system established by [16]. This categorized them as poor, medium, or good. Animals recorded as dewormed had been given antiparasitic drugs (ivermectin or albendazole) within 3–6 months of this work [17].

### 2.5. Sample Collection and Laboratory Analysis

A fecal sample was collected directly from the rectum of each animal. It was placed in labeled containers, and these labels were correlated with prepared questionnaires that included details about the independent variables. These samples were transported to the veterinary parasitology laboratory at Addis Ababa University. The sample was placed in the refrigerator until laboratory processing.

At the laboratory, a qualitative method using a flotation technique was employed for the detection of strongyle eggs [18]. Around 3 g of the fecal sample was measured, placed into a mortar, and crushed into small pieces with a pestle before being transferred into a glass beaker, where a saturated sodium chloride solution was added. Then, the mixture was stirred continuously by using a glass rod. The dissolved suspension was strained into another beaker using a tea strainer and gauze, which was followed by the transfer of the suspension into a test tube up to meniscus formation at the top of the tube. A cover slip was placed over the meniscus gently and allowed to stand for 20 min. In the end, the coverslip was lifted off from the test tube gently and was observed under a microscope at low magnification power (10×).

For quantitative analysis, Stoll's egg counting method was used to estimate the EPG of feces, which indicates the parasite burden. In this method, the sensitivity is such that one egg observed corresponds to 100 EPG. All laboratory procedures were carried out within 24 h of collection to ensure accuracy and reliability of results [19].

Here's how the calculation works:

Four grams of feces + 56 mL of water = total volume = 60 mL ⇒ the feces were diluted 15 times (60 mL/4 g). A 0.15 mL aliquot was examined under the microscope, and 0.15 mL was 1/400 of the total 60 mL (60 mL/0.15 mL = 400). Since 4 g of feces was diluted and 1/400 of it was examined under a microscope, multiply the number of eggs seen by (60/0.15) × (1/4) = 100.

Finally, fecal culture was employed to harvest their infective larval stage (L3). In this stage, the parasite was differentiated on the morphological characteristics of L3 larvae [24; 25].

### 2.6. Data Management and Analysis

The collected raw data was verified for its accuracy and completeness. The completed data were recorded by using a Microsoft Excel Version 2021 spreadsheet and imported into Stata Version 14 statistical software for data management and analysis. The combination of descriptive (proportion and mean) and inferential statistics (chi-squared and logistic regression) was employed to examine the relationship between independent variables with *H. contortus* and other strongyle egg counts. Univariable and multivariable binary logistic regressions were used to assess the risk factors for *H. contortus* and other strongyle infections. Univariable logistic regression was applied to compute the crude odds ratio and to screen candidate variables for final model development. Multivariable binary logistic regression was performed to compute the adjusted odds ratio and to account for the effect of confounding. The fitness of the final model was checked by using the Hosmer–Lemeshow test. The Mann–Whitney test (in two groups) and the Wilcoxon rank sum test (in three or more groups) were also employed to compare the mean EPG of strongyle eggs between different factors when the assumption of the corresponding parametric statistical test was not satisfied. For all data analyses, a *p* value less than 0.05 was considered statistically significant.

## 3. Result

### 3.1. Overall Prevalence of Haemonchosis

A total of 384 small ruminants, of which 262 (68.2%) were sheep, were recruited randomly to estimate the prevalence of *H. contortus* and other strongyle infection from the presence of eggs in fecal samples. A total of 278 (72.4%) small ruminants were positive for strongyle-type egg. Of those animals positive for strongyle infection, the prevalence of *H. contortus*, as found by coproculture, was 66.9% (186 animals), with prevalence higher in sheep (73.3%) than goats (53.3%).

As Table 1 illustrates, the prevalence of was highest in Dhibayu, among younger animals, females, and in extensively farmed sheep. Flock size and type also played a role, with higher prevalence in larger flocks and those flocks containing only sheep. Also, the source of drinking water mattered, with shoats drinking from rivers having a higher infection rate than those on tap water.

The result of the chi-square test depicted that the variables species, age, sex, management, flock type, and water source had statistically significant associations (*p* value < 0.05) with the prevalence of *H. contortus* infection in small ruminants, whereas origin, body condition, and flock size had not (*p* value > 0.05) (Table 1).

### 3.2. Risk Factors for *H. contortus* Prevalence

Variable screening was conducted by binary logistic regression (crude odds ratio) analysis of each independent variable with *H. contortus* detected in small ruminants at a 25% level of significance. From the crude analysis, seven factors, such as species, management, flock type, origin, water source, age, and sex, had statistically significant associations (*p* value < 0.25) with the prevalence of *H. contortus* in small ruminants. However, body condition, deworming history, and flock size were not statistically significant (*p* value > 0.25). On the other hand, management and water source had multicollinearity for *H. contortus* so that water source was removed from the multivariable analysis. Therefore, six factors were included, and conducted multivariable analysis was conducted. Eventually, four covariates, such as semiextensive management, young age group, female sex, and sheep species, were found to be statistically significant (*p* value < 0.05) factors for *H. contortus* in small ruminants at 5% level of significance (Table 1).

Species were a statistically significant risk factor for the prevalence of *haemonchosis*. For sheep, the odds of developing *H. contortus* increase by 2.80-fold compared to goats by holding other variables in the model constant (AOR = 2.80, 95% CI = 1.54–3.77, *p* = 0.035). Age was another statistically significant risk factor for the prevalence of haemonchosis. For adults, the odds of developing *Haemochosis* decrease by 0.39-fold compared to young shoats by holding the other variables in the model constant (AOR = 0.39, 95% CI = 0.23–0.69, *p* = 0.001). Similarly, sex was also a statistically significant risk factor for the prevalence of *haemonchosis*. For males, the odds of developing *Haemonchus* infection decrease by 0.43-fold compared to females by holding the other variables in the model constant (AOR = 0.43, 95% CI = 0.27–0.69, *p* = 0.001). Management was also another significant risk factor for the occurrence of haemonchosis. Small ruminants found in semi-intensive management, the odds of developing *Haemonchus* infection decrease by 63% compared to extensive management by holding the other variables in the model constant (AOR = 0.37, 95% CI = 0.21–0.63, *p* = 0.001).

### 3.3. Result of Quantitative Fecal Egg Count

Fecal samples that were positive for the flotation technique were subjected to Stoll's method of egg counting to determine the degree of severity of parasitic infection. The study investigated the association between fecal egg counts (EPG) of strongyle parasites and potential risk factors. Although factors like origin, flock size, flock type, and deworming history were not significantly linked to EPG levels (*p* > 0.05), species, sex, age, body condition, and management did show a statistically significant association (*p* < 0.05) with EPG counts (Table 2).

**Figure 2:**
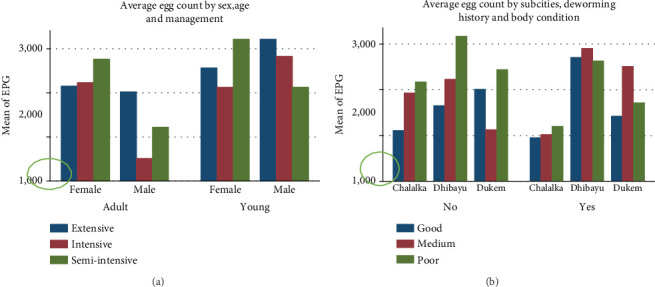
(a) Average egg count by sex, age, and management system and (b) average egg count by subcities, deworming history, and body condition.

Furthermore, the study examined average EPG values for each risk factor. The average egg count in young and female animals managed in extensive and semi-intensive management systems was slightly higher than in intensively managed animals (Figure 2a). Shoats from Dhibayu had the highest average EPG (2566.07) compared to those from Chalalka (1402.48) and Dukem (1816.84). Similarly, shoats that had not been dewormed had a slightly higher average EPG (2088.39) compared to those that had been dewormed (1910) (Figure 2b and Table 3).

The Kruskal–Wallis test depicted that the predictor's origin and management had statistically significant (*p* value < 0.05) differences between the mean EPG of *strongyle* eggs in shoats, whereas body condition, flock type, and flock size did not (*p* value > 0.05). On the other hand, the Mann–Whitney test portrayed that sex, species, and age had statistically significant (*p* value < 0.05) differences between the mean EPG of *strongyle* eggs in shoats, whereas deworming history did not (*p* value > 0.05) (Table 3).

## 4. Discussion

The coprological examination conducted in this study showed an overall prevalence of 66.9% (95% CI: 62.0–71.5) for *H. contortus* infection in small ruminants in Bishoftu, Ethiopia. This is a higher prevalence than previously reported in Ethiopia: 26.8% prevalence at Abergele abattoir in Mekele [20], 33.1% in Jimma [21], 31.5% in and around Nekemte town [17], 46.1% in Mitto District [22], 40.4% in and around Haramaya [23], 40.9% in Wukro [24], and 55% in and around Haramaya [25].

On the other hand, the present study outcome is lower than the result of the prevalence of *H. contortus* infection in Gonder, 80.2% and 73.6%, respectively [6; 32]; 77.4%, 95.8%, and 87.1% report on the prevalence of abomasal nematode in Bishoftu, Haramaya, and Ogaden, respectively [7; 33; 34]. Outside of Ethiopia, there is some variation in reports of the prevalence of *H. contortus* infection: 75.5% in sheep and goats in Nyagatare District, Rwanda [26], 57.8% in Bangladesh [27], 80.3% in Nigeria [28], 77.7% in Pakistan [29], and 23.9% in Morocco [30].

This variation in the prevalence of *H. contortus* infection varies across regions and countries due to several factors. This includes differences in agroclimatic conditions such as environmental conditions of humidity, temperature, and rainfall affecting pastures that could support extended survival and development of infective larval stage of *H. contortus*, study sample sizes, management system of examined animals, characteristics of breeds themselves, number of animals graze together, presence or absence of intercurrent infections, availability of veterinary care, and local knowledge about managing animals and treating the parasite including anthelmintic usage. All these factors influence the parasite's life cycle and spread throughout different areas [10; 26].

A statistical analysis revealed a higher prevalence of *H. contortus* infection in sheep compared to goats, suggesting greater susceptibility in sheep. The result of this study is consistent with other reports: 63.6% in sheep and 61.8% in goats in Ejere, West Shoa [7]; 90.1% in sheep and 81.8% in goats at Haramaya municipal abattoir, eastern Hararghe [31]; 52.1% in sheep and 33.1% in goats in Mitto District [22]; 91.2% in sheep and 82.9% in goats in Ogaden region, eastern Ethiopia [32]; 65.6% in sheep and 8% in goats in and around Gondar [8]; and 69.6% in sheep and 57.1% in goats [24]. However, this finding contradicts those of a previous study, 67.57% in sheep and 71.39% in goats in and around Finoteselam [33]. The higher prevalence of *H. contortus* infection in sheep compared to goats could be explained by their grazing habit. Sheep tend to graze closer to the ground, where infective larvae of the parasite (L3) are most concentrated on contaminated plants. Goats, on the other hand, browse bushes and trees, which are likely out of reach for these larvae, making them less likely to get infected [31; 13].

Female sheep were more likely to be infected with *H. contortus* compared to males, consistent with the report 51.4% in females and 36.3% in males in Mitto District [22]. This difference in females may be due to greater nutritional demands and/or hormonal fluctuations experienced by females throughout their reproductive cycle, lactation, and parturition, potentially weakening their immune system and making them more susceptible to parasites [11; 29]. However, these findings on sex differ from others. For instance, Zelalem et al. [33] reported higher infection rates in both males (73.22%) and females (64.71%) near Finoteselam, Amhara. Similarly, Lidya and Tadele [34] reported a higher prevalence in males (29.7%) than in females (11.2%) in Wukro.

This report revealed younger sheep (< 1 year) to be more susceptible to *H. contortus* infection compared to adults (> 1 year), in agreement with previous research by [24] who reported similar results in 66.9% of young and 59.0% of adults in Bishoftu, Ethiopia. But Zelalem et al. [33] indicated that the prevalence in young and adult sheep was 67.50% and 71.43%, respectively. This higher prevalence in young sheep is likely their immune systems being less developed, due to their lack of prior exposure to the parasite. Adults, on the other hand, have likely developed some immunity through repeated exposure over time. This age-related resistance helps explain the lower infection rates observed in elder animals [22].

The parasite load in the animal was determined in 384 sampled sheep and goats; 29.2%, 12.1%, and 58.8% were infested lightly, moderately, and severely, respectively. This result differs from other studies, with 9.2% (massively), 25.2% (moderately), and 65.6% (lightly) in Gechi District [35] and 50% (lightly), 25% (moderately), and 20% (heavily) in Kuarit District [36]. Additionally, the study explored potential links between the severity of infection and association factors.

The current report showed that the prevalence of the *Haemonchus* parasite was similar among animals originating from three subcities of the study areas, with higher prevalence in sheep and goats originating from Dhibayiu (72.02%) than Chalalka (65.29%) and lastly Dukem (60.00%), with no statistical difference (*p* value > 0.05). This might be due to the similar environmental factors (temperature and humidity) in which the subsites reside in the same agroecology.

## 5. Conclusion

The findings of this study indicate a high prevalence of *H. contortus* infection and other strongyle parasites among sheep and goats in Bishoftu, with prevalence significantly influenced by species, sex, and age of animals. Sheep exhibited a higher prevalence (73.3%), compared to goats (53.3%), and females (74.8%) were more affected than males (58.6%). Young animals showed a markedly higher infection rate (78.5%) than adults (62.5%). These results underscore the need for strategic and targeted parasite control measures, particularly focusing on the most susceptible groups—young and female animals, as well as species-specific interventions. Therefore, it is important to implement targeted and seasonally based deworming strategies using effective anthelmintics, particularly before periods of high parasite transmission. This approach should be based on local epidemiological data to reduce parasite resistance and emphasize the susceptibility of young and female sheep and goats.

## Figures and Tables

**Table 1:** Prevalence of *H. contortus* infection by univariable and multivariable binary logistic regression analysis in small ruminants (*n* = 384) in Bishoftu, Ethiopia.

**Risk factors**	**Categories**	**No examined**	**COR (95% CI COR)**	**Crud** **p** ** value**	**AOR 95% (CI AOR)**	**Adjusted** **p** ** value**
Species	Goat	122	2.41 (1.54–3.77) Ref	0.001	2.80 (1.24–6.38)	0.035
	Sheep	262		Ref	Ref	

Origin	Chalalka	121	0.73 (0.44–1.21)	0.222	0.88 (0.49–1.55)	0.653
	Dukem	95	0.58 (0.34–0.99) Ref	0.046	0.71 (0.39–1.28)	0.250
	Dhibayu	168		Ref	Ref	

Sex	Male	198	0.48 (0.31–0.74) Ref	0.001	0.43 (0.27–0.69)	0.001
	Female	186		Ref	Ref	

Age	Adult	277	0.46 (0.27–0.77) Ref	0.003	0.39 (0.23–0.698)	0.001
	Young	107		Ref	Ref	

Body condition	Medium	122	0.88 (0.53–1.46)	0.613		
	Poor	142	1.15 (0.68–1.95) Ref	0.591		
	Good	120		Ref		

Management	Intensive	168	0.32 (0.19–0.53)	0.001	0.75 (0.36–1.57)	0.448
	Semi-intensive	63	0.74 (0.38–1.46) Ref	0.392	0.37 (0.21–0.63)	0.001
	Extensive	153		Ref	Ref	

Flock size	Medium	54	0.90 (0.34–2.39)	0.833		
	Semilarge	98	1.39 (0.55–3.498)	0.482		
	Large	206	1.00 (0.43–2.38) Ref	0.988		
	Small	26		Ref		

Flock type	Mixed	161	1.66 (0.97–2.83)	0.065	0.47 (0.19–1.15)	0.100
	Only sheep	136	2.34 (1.32–4.15) Ref	0.003	0.60 (0.21–1.698)	0.337
	Only goat	87		Ref	Ref	Ref

Deworming history	Dewormed	160	0.82 (0.53–1.26)	0.369		

Abbreviations: A*β*, adjusted coefficients; AOR, adjusted odds ratio; CI, confidence interval; COR, crude odds ratio.

∗Statistical significance.

**Table 2:** Extent of strongyle infection associated with different risk factors in sheep and goats in Bishoftu, Ethiopia.

**Risk factors**	**Categories**	**Degree of infection**			**X** ^2^	**p** **value**
		**Light%**	**Moderate%**	**Heavy%**		
Species	Sheep	60 (80%)	24 (77.42%)	108 (71.52%)	16.83	0.001
	Goat	15 (20%)	7 (22.58%)	43 (28.48%)		

Origin	Dhibayu	30 (40%)	14 (45.16%)	77 (50.99%)	9.02	0.173
	Chalalka	30 (40%)	10 (32.26%)	39 (25.83%)		
	Dukem	15 (20%)	7 (22.58%)	35 (23.18%)		

Sex	Female	39 (52%)	16 (51.61%)	93 (61.59%)	13.64	0.003
	Male	36 (48%)	15 (48.39%)	58 (38.41%)		

Age	Young	22 (29.3%)	7 (22.58%)	55 (36.42%)	12.03	0.007
	Adult	53 (70.7%)	24 (77.42%)	96 (63.58%)		

Body condition	Poor	17 (22.67%)	17 (54.84%)	61 (40.40%)	13.16	0.041
	Medium	26 (34.67%)	7 (22.58%)	45 (29.80%)		
	Good	32 (42.67%)	7 (22.59%)	45 (29.80%)		

Management	Intensive	25 (33.33%)	12 (38.71%)	54 (35.76%)	23.47	0.001
	Semi-intensive	12 (16.00%)	6 (19.35%)	28 (18.54%)		
	Extensive	38 (50.67%)	13 (41.94%)	69 (45.70%)		

Flock size	Small	6 (8.00%)	1 (3.23%)	10 (6.62%)	7.05	0.632
	Medium	12 (16.00%)	4 (12.90%)	18 (11.92%)		
	Semilarge	18 (24.00%)	13 (41.94%)	40 (26.49%)		
	Large	39 (52.00%)	13 (41.94%)	83 (54.97%)		

Flock type	Only sheep	31 (41.33%)	14 (45.16%)	56 (37.09%)	11.01	0.088
	Mixed	31 (41.33%)	14 (45.16%)	63 (41.72%)		
	Only goat	13 (17.33%)	3 (9.68%)	32 (21.19%)		

Deworming history	Dewormed	30 (40.00%)	14 (45.16%)	59 (39.07%)	1.19	0.753
	Not deworm	45 (60.00%)	17 (54.84%)	92 (60.93%)		

**Table 3:** Average fecal worm egg count in strongyle positive animals with respective risk factors from flocks and herds in Bishoftu, Ethiopia.

**Risk factors**	**Category**	**Mean**	**[95% CI]**	**χ** ^2^	**p** **value**
Species	Sheep	2139	1773.74–2505.65	2.48	0.013
	Goat	1744	1266.75–2221.65		

Origin	Dhibayu	2566	2045.66–3086.48	7.92	0.019
	Chalalka	1402	1027.36–1777.60		
	Dukem	1816	1282.94–2350.74		

Sex	Female	2322	1920.48–2723.97	3.53	0.001
	Male	1686	1263.99–2108.05		

Age	Young	2677	2044.30–3310.84	3.33	0.001
	Adult	1757	1438.89–2076.63		

Body condition	Poor	2354	1853.43–2866.62	2.25	0.325
	Medium	1904.	1406.21–2401.99		
	Good	1722	1222.95–2222.05		

Management	Intensive	1663	1231.97–2094.22	14.34	0.001
	Semi-intensive	2166	1491.02–2842.31		
	Extensive	2336	1853.43–2819.78		

Flock size	Small	1892	929.95–2854.66	2.69	0.442
	Medium	1546	927.29–2165.31		
	Semi-large	2294	1705.373–2884.42		
	Large	2018	1597.17–2439.72		

Flock type	Only sheep	2169	1672.08–2666.15	3.44	0.179
	Mixed	1970	1513.17–2427.21		
	Only goat	1852	1259.37–2446.38		

Deworming history	Dewormed	1910	1475.56–2344.44	0.91	0.362
	Not deworm	2088	1694.22–2482.58		

## Data Availability

The data that support the findings of this study are available from the corresponding author upon reasonable request.
